# Effects of in ovo injection of the L-carnosine on physiological indexes of neonatal broiler chicken

**DOI:** 10.1016/j.psj.2023.103380

**Published:** 2023-12-19

**Authors:** Mahta Keshtkaran, Shahin Hassanpour, Kaveh Parvandar Asadollahi, Morteza Zendehdel

**Affiliations:** ⁎ Faculty of Veterinary Medicine, Science and Research Branch, Islamic Azad University, Tehran, Iran; † Division of Physiology, Department of Basic Sciences, Faculty of Veterinary Medicine, Science and Research Branch, Islamic Azad University, Tehran, Iran; ‡ Department of Clinical Sciences, Faculty of Veterinary Medicine, Science and Research Branch, Islamic Azad University, Tehran, Iran; § Department of Basic Sciences, Faculty of Veterinary Medicine, University of Tehran, 14155-6453 Tehran, Iran

**Keywords:** in ovo injection, L-carnosine, chicken

## Abstract

The objective of the present investigation was to ascertain the impact of in ovo administration of L-carnosine on physiological indicators in neonatal broiler chickens. A total of 280 viable broiler eggs were allocated to 7 distinct groups: control, Sham in ovo injection of sterile water on d 7 of incubation. Groups 3 and 4 were subjected to in ovo injections of L-carnosine (25 and 50 µg) on d 7 of incubation. Group 5, functioning as a sham in ovo, received an injection of sterile water on d 18 of incubation. Groups 6 and 7 were in ovo injected with L-carnosine (25 and 50 µg) on d 18 of incubation. All eggs were subjected to incubation, and the hatching rate and body weight were measured post-hatch. Subsequently, blood samples were collected, and the levels of biochemical constituents in the serum were determined. Based on the outcomes, the administration of L-carnosine (50 µg) on d 7 of incubation led to a significant increase in post-hatch body weight compared to the control group (*P* < 0.05). The in ovo injection of L-carnosine (25 and 50 µg) on d 7 and 18 of incubation resulted in a significant decrease in the levels of serum glucose, triglyceride (**TG**), low-density lipoprotein (**LDL**), phosphorus (**P**), alkaline phosphatase (**ALP**), aspartate aminotransferase (**AST**), and alanine transaminase (**ALT**) in the newly hatched chickens (*P* < 0.05). Furthermore, the in-ovo injection of L-carnosine (25 and 50 µg) on d 7 and 18 of incubation led to a significant increase in the levels of serum high-density lipoprotein (**HDL**), calcium, and total protein (**TP**) in the newly hatched chickens (*P* < 0.05). Nonetheless, L-carnosine did not have a significant effect on the levels of serum IgY and IgA in the newly hatched chickens (*P* > 0.05). These findings indicate that the in ovo administration of L-carnosine yielded favorable outcomes in neonatal broiler chickens.

## INTRODUCTION

The complexity of growth physiology is evident throughout the developmental stages, ranging from embryonic to neonatal. Moreover, it is worth noting that the growth pattern in avian species differs somewhat from that observed in mammals (Teymouri et al., 2020). Embryonic development in chickens occurs within the confines of the egg, functioning as a semiclosed system that provides essential nutrients for development, with only gas and water exchange taking place (Cherian, 2022). Toward the latter stages of development, there is an increased demand for energy to facilitate rapid growth. However, the growth rate becomes compromised due to limited nutrient and energy availability (Nasri et al., 2020). In commercial poultry production systems, it is common for food and water availability to be delayed for 24 to 48 h posthatch during transportation between the hatchery and the production farm. This delay negatively impacts the early feed intake and development of chickens (Nouri et al., 2018). In an effort to enhance prehatch and post-hatch outcomes, early feeding through in ovo inoculation has been developed to deliver nutrients and feed additives during the embryonic stage (Arain et al., 2022). Extensive research has been conducted on the delivery of supplements, nutrients, drugs, and vaccines through the in ovo feeding system. The results have consistently demonstrated that feeding during the embryonic stage improves weight gain, feed efficiency, growth rate, immunity, and overall health, while reducing morbidity and mortality rates. Additionally, it enhances muscular development and meat yield in poultry (Han et al., 2020).

L-Carnosine, a dipeptide synthesized endogenously from L-histidine and β alanine, is abundantly present in mammalian skeletal muscles and the nervous system. This compound possesses significant physiological properties, including protein protection, anticonvulsive and antioxidant effects, as well as the regulation of Ca^2+^ sensitivity (Schwank-Xu et al., 2021). It has been reported that the administration of L-carnosine leads to increased levels of insulin and insulin-like growth factor 1, while simultaneously decreasing blood glucose levels (Peters et al., 2020). Furthermore, L-carnosine has the ability to reduce neuronal amyloid-β levels, macrophage nitric oxide (**NO**) production, and cognitive deficits (Caruso et al., 2019). Its benefits extend to brain and lung maturation, as well as fetal growth (Arenas et al., 1998). In mice offspring, maternal consumption of L-carnosine has been found to increase body weight and enhance renal oxidative status (Nguyen et al., 2015). Additionally, L-carnosine exerts neuroprotective effects on cortical neurons and mitigates inflammation and degeneration by suppressing the synthesis of tumor necrosis factor-β (**TNF-β**) (Tsuji et al., 2022).

The provision of additional nutrients through in ovo feeding has been shown to enhance the growth of embryos by overcoming any limitations in egg nutrition and providing a greater abundance of nourishment (Teymouri et al., 2020). The presence of L-carnosine in the diet has been found to possess antioxidant properties, and the inclusion of dietary carnosine at a concentration of 0.50% during either d 1 to 21 or 21 to 42 has been demonstrated to improve both the quantity and quality of chicken meat. Furthermore, it has been observed that the consumption of carnosine leads to a reduction in the levels of thiobarbituric acid reactive substances and an increase in the overall antioxidant capacity within the blood and muscle (Hu et al., 2009). The supplementation of carnosine and L-histidine in the chicken diet has also been shown to elevate the carnosine content within the breast muscles of chickens (Kopec et al., 2020). Likewise, Cong et al. (2017) reported in a similar study that the inclusion of carnosine at levels of 100, 200, or 400 mg/kg in the diet enhances meat quality and antioxidant capacity, while simultaneously decreasing lipid peroxidation in breast meat. The utilization of L-carnitine in poultry feed has been found to enhance energy efficiency, enabling poultry to more effectively acquire the necessary energy from dietary lipids. The inclusion of L-carnitine in the diet has been associated with improvements in energy efficiency, body weight gains, and feed conversion ratio in poultry. Additionally, L-carnitine has been observed to enhance the antioxidant and immune capacity of poultry (Azizi-chekosari et al., 2021).

Despite the existing body of research on the impact of dietary L-carnosine on poultry performance and productivity, no studies have been conducted on its application as an in ovo inoculation. Consequently, the objective of the present investigation was to assess the effects of in ovo injection of l-carnosine on the physiological parameters of neonatal broiler chickens.

## MATERIALS AND METHODS

### Animals

**Figure 1:**
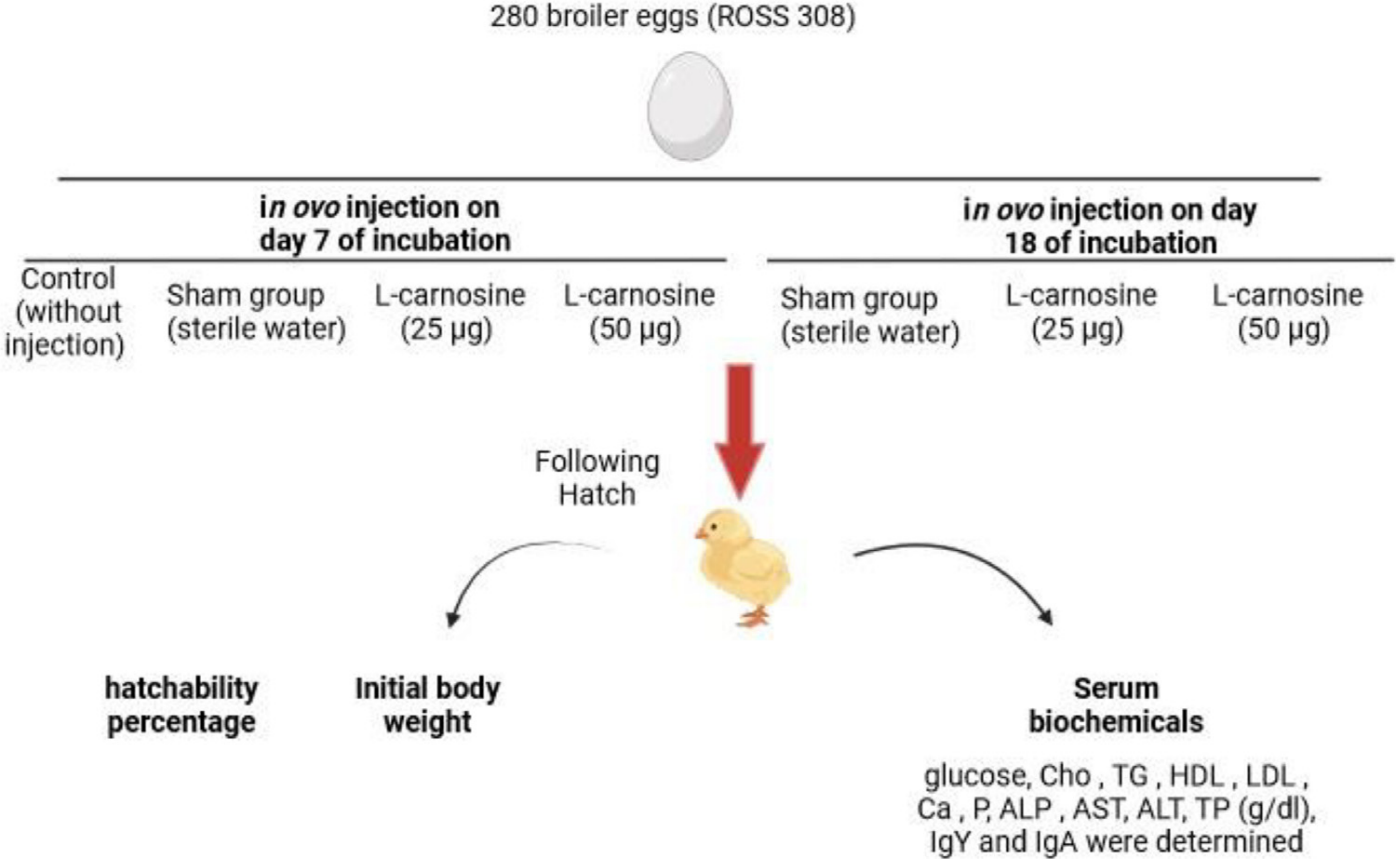
Flow chart of study.

Two hundred and eighty fertile broiler eggs (ROSS 308) were acquired from a local hatchery (Morghak Co., Iran). All eggs were obtained from the same breeder flock with an average age of 36 to 37 wk. The eggs were divided into 7 experimental groups with 40 eggs in each group: the control group (without injection) and the sham group (in ovo injection of sterile water on d 7 of incubation). Group 3 received an in ovo injection of L-carnosine (25 µg, Sigma-Aldrich) on d 7 of incubation. Group 4 received an in ovo injection of L-carnosine (50 µg) on d 7 of incubation. Group 5 served as the sham group and received an in ovo injection of sterile water on d 18 of incubation. Group 6 received an in ovo injection of L-carnosine (25 µg) on d 18 of incubation, and group 7 received an in ovo injection of L-carnosine (50 µg) on d 18 of incubation (Ghiasi Ghalehkandi et al., 2012) (Figure 1). The eggs were candled twice during incubation to eliminate infertile eggs and eggs with deceased embryos. The research committee at the Islamic Azad University, Science and Research Branch (IR IAU.SRB.REC.1401.008) approved all study protocols.

### In Ovo Feeding Procedure

The in ovo feeding procedure was conducted on d 7 and 18 of incubation. The procedure involved injecting the eggs through the blunt end (Uni et al., 2005) at a temperature of 37°C. The injection point on the eggshell indicated the air cell position. Prior to injection, the injection point was sterilized with 70% ethanol. A hole was created using a dental drill bit, ensuring the chorioallantoic membrane was not penetrated (Uni et al., 2005). The injection was performed using a 22G needle in the albumen, and the area was disinfected with an ethyl alcohol-laden swab and sealed with cellophane tape. Each needle was used for a single injection. The eggs were placed on plastic flats and cooled at a temperature of 18.6°C with a humidity level of 75% at room temperature. Random incubation of the eggs was carried out in an automatic incubator (37.2°C, humidity 60%) and transferred to industrial hatchers on d 18 (37.5°C, airspeeds 0.2 m/s, humidity 75%) (Uni et al., 2005).

### Blood Biochemical Analysis

After hatching, the hatchability percentage and initial body weight were determined. Three broilers from each group were randomly selected, and blood samples were collected. The blood samples (2 mL) were centrifuged at 250 × *g* for 10 min to separate the serum, which was then stored at −20°C until further use. The serum was used to measure glucose (mg/dL), Cho (mg/dL), TG (mg/dL), HDL (mg/dL), LDL (mg/dL), Ca (mg/dL), P (mg/dL), ALP (IU/L), AST (IU/L), ALT (IU/L), TP (g/dL), IgY (mg/mL), and IgA (mg/mL).

### Statistical Analysis

The obtained data were subjected to 1-way analysis of variance (**ANOVA**) and presented as the mean ± standard error (**SE**). Mean values of treatments showing differences were compared using the Tukey HSD test (*P* < 0.05).

## RESULTS

The data presented in the Table 1 demonstrate that the administration of L-carnosine (at a dose of 25 µg) through in ovo injection on the seventh day of incubation, as well as the injection of distilled water on the 18th day, resulted in a significant reduction in hatchability when compared to the control group (*P* < 0.05). However, no significant difference in hatchability was observed when the L-carnosine was injected at a dose of 50 µg on the seventh day or on the 18th day of incubation, in comparison to the control group (*P* < 0.05). Moreover, the in ovo injection of L-carnosine (at a dose of 50 µg) on the seventh day of incubation led to a significant increase in post-hatch body weight when compared to the control group (*P* < 0.05). However, there was no significant difference in body weight observed when the L-carnosine was injected on the 18th day of incubation, in comparison to the control group (*P* < 0.05).

**Table 1:** Effects of in ovo injection of the L-carnosine on different days of incubation on hatchability and serum biochemical in post hatched broiler chicken.

	D 7 of incubation				D 18 of incubation		
	Control	Sham group (sterile water)	L-carnosine (25 µg)	L-carnosine (50 µg)	Sham group (sterile water)	L-carnosine (25 µg)	L-carnosine (50 µg)
Hatchability (%)	94 ± 8.36^a^	86 ± 8.64^a^	74.5 ± 6.55^b^	55 ± 6.12^c^	97 ± 7.98^a^	87.5 ± 7.45^a^	89 ± 8.34^a^
Initial body weight (g)	38.01 ± 2.98^b^	37.30 ± 2.37^b^	38.20 ± 2.87^b^	42.90 ± 3.79^a^	38.00 ± 2.99^b^	41.48 ± 4.69^a^	43.91 ± 4.34^a^
Glu (mg/dL)	265.62 ± 10.12^a^	263.21 ± 10.45^a^	218.88 ± 10.05^b^	204.06 ± 10.32^b^	253.18 ± 11.23^a^	210.28 ± 10.10^b^	155.30 ± 9.68^c^
TG (mg/dL)	73.44 ± 4.34^a^	72.55 ± 4.21^a^	65.04 ± 4.12^b^	64.80 ± 3.89^b^	48.60 ± 3.21^c^	43.72 ± 3.65^c^	33.44 ± 3.11^d^
HDL (mg/dL)	122.32 ± 9.87^c^	119.36 ± 9.65^c^	130.54 ± 10.26^b^	165.84 ± 10.32^a^	130.25 ± 9.20^c^	141.25 ± 10.41^b^	163.20 ± 10.55^a^
LDL (mg/dL)	92.12 ± 4.30^a^	90.21 ± 3.65^a^	92.54 ± 4.12^a^	75.45 ± 3.74^b^	91.69 ± 5.14^a^	94.21 ± 5.10^a^	68.77 ± 3.94^b^
Ca (mg/dL)	10.21 ± 1.41^c^	10.36 ± 1.36^c^	11.11 ± 1.10^b^	13.49 ± 1.54^a^	10.75 ± 1.06^c^	11.86 ± 1.12^b^	14.82 ± 1.02^a^
P (mg/dL)	4.57 ± 0.65^a^	4.61 ± 0.59^a^	4.50 ± 0.46^a^	3.91 ± 0.29^b^	4.15 ± 0.36^b^	3.82 ± 0.34^b^	3.99 ± 0.29^b^
ALP (IU/L)	3355 ± 157.69^b^	3500 ± 151.32^b^	2456 ± 146.35^c^	1437 ± 131.98^d^	3031 ± 140.23^c^	4258 ± 120.50^a^	4093 ± 131.26^a^
AST (IU/L)	233.5 ± 12.32^a^	234 ± 13.11^a^	223.3 ± 12.65^a^	146.1 ± 10.47^d^	192.1 ± 10.21^b^	173.8 ± 10.45^c^	154.8 ± 10.69^d^
ALT (IU/L)	4.17 ± 0.87^a^	4.12 ± 0.84^a^	2.73 ± 0.54^b^	1.39 ± 0.20^c^	4.17 ± 0.91^a^	2.78 ± 0.49^b^	1.51 ± 0.24^c^
TP (g/d)	1.78 ± 0.32^d^	1.73 ± 0.29^d^	2.16 ± 0.51^c^	2.58 ± 0.81^b^	2.12 ± 0.52^c^	2.78 ± 0.84^b^	3.28 ± 0.98^a^
IgY (mg/mL)	7.10 ± 1.12	7.00 ± 1.02	7.10 ± 0.98	7.20 ± 1.04	7.10 ± 0.97	7.08 ± 1.21	7.08 ± 0.89
IgA (mg/mL)	0.44 ± 0.05	0.43 ± 0.04	0.39 ± 0.03	0.44 ± 0.040	0.42 ± 0.050	0.39 ± 0.03	0.41 ± 0.04

Glu: glucose, TG: triglyceride, HDL: high density lipoprotein, LDL: low density lipoprotein, Ca: calcium, P: phosphorus, ALP: alkaline phosphatase, AST: aspartate aminotransferase, ALT: alanine transaminase, TP: total protein, IgY: immunoglobulin Y, IgA: IgY immunoglobulin Y. In each row, there are significant differences between groups with different superscripts (a–d; *P* ≤ 0.05).

Based on the findings, it can be concluded that the in ovo injection of L-carnosine (at doses of 25 µg and 50 µg) on the seventh and 18th days of incubation resulted in a significant decrease in serum glucose levels in newly hatched chickens when compared to the control group (*P* < 0.05). Additionally, a significant difference was observed between the experimental groups on the 18th day of incubation, with the injection of L-carnosine at a dose of 50 µg leading to a greater reduction in serum glucose levels compared to the injection of 25 µg of L-carnosine in newly hatched chickens (*P* < 0.05). In this study, it was found that the in ovo injection of L-carnosine (at doses of 25 µg and 50 µg) on the seventh day of incubation resulted in a significant decrease in serum TG levels in newly hatched chickens when compared to the control group (*P* < 0.05). Additionally, the in ovo injection of L-carnosine at a dose of 50 µg on the 18th day of incubation also led to a significant reduction in serum TG levels in newly hatched chickens when compared to the control group (*P* < 0.05).

As observed, the in ovo administration of L-carnosine (25 and 50 µg) on the seventh day of incubation resulted in a significant elevation of serum HDL in newly hatched chickens, when compared to the control group (*P* < 0.05). Furthermore, the in ovo injection of L-carnosine (25 and 50 µg) on the 18th day of incubation significantly enhanced serum HDL in newly hatched chickens in comparison to the control group (*P* < 0.05). Notably, the level of 50 µg L-carnosine had a significantly greater effect on HDL levels than 25 µg L-carnosine on both injection days (*P* < 0.05). In this particular study, the in ovo injection of L-carnosine (50 µg) on the seventh and 18th day of incubation led to a significant decrease in serum LDL levels in newly hatched chickens, when compared to the other groups (*P* < 0.05). However, there was no significant difference observed between the other groups and the control group in terms of serum LDL levels in newly hatched chickens (*P* > 0.05).

As observed, the in ovo injection of L-carnosine (25 and 50 µg) on the seventh and 18th day of incubation significantly increased serum calcium levels in newly hatched chickens, when compared to the control group (*P* < 0.05). Notably, the level of 50 µg L-carnosine had a significantly greater effect on serum calcium levels than 25 µg L-carnosine on both injection days (*P* < 0.05). In this study, the in ovo injection of L-carnosine (50 µg) on the seventh day of incubation significantly reduced serum phosphorus levels in newly hatched chickens, when compared to the control group (*P* < 0.05). However, no significant difference was observed between the other injected groups and the control group in terms of serum phosphorus levels in newly hatched chickens (*P* > 0.05).

As observed, in ovo injection of the L-carnosine on d 7 of incubation significantly reduced serum ALP levels in newly hatched chicken compared to the control group (*P* < 0.05). In contrast, different levels of the L-carnosine on d 18 of incubation significantly elevated serum ALP levels in newly hatched chicken (*P* < 0.05). Based on results in Table 1, in ovo injection of the L-carnosine (50 µg) significantly diminished serum AST in comparison to control chicken (*P* < 0.05). Additionally, L-carnosine (25 and 50 µg) on d 18 of incubation significantly declined serum AST than the control chicken (*P* < 0.05). According to results, in ovo injection of the L-carnosine on both days significantly decreased serum APT compared to control chicken (*P* < 0.05). As shown, in ovo injection of the L-carnosine in both days of the incubation y a dose-dependent manner increased serum TP levels in newly hatched chicken (*P* < 0.05). As seen, in ovo injection of the L-carnosine had no significant effect on serum IgY levels of the newly hatched chicken (*P* < 0.05). Also, in ovo injection of the L-carnosine had no significant effect on serum IgA levels of the newly hatched chicken (*P* < 0.05).

## DISCUSSION

In the present investigation, we have successfully demonstrated, for the very first time, the efficacy of in ovo administration of L-carnosine in broilers. The outcomes of this study indicate that the in ovo provision of L-carnosine leads to a notable enhancement in both hatchability and post-hatch body weight. Furthermore, the in ovo injection of L-carnosine results in a reduction of serum glucose, TG, LDL, P, ALP, AST, and ALT levels in newly hatched chickens. Additionally, this injection promotes elevated levels of serum HDL, Ca, and TP in the same group of chickens. However, it is worth noting that L-carnosine does not exhibit any significant impact on the immune system serum, specifically with regards to IgY and IgA. L-carnosine is a naturally occurring antioxidant and possesses a multitude of physiological functions. In mammals, a staggering 99% of L-carnosine is concentrated within the skeletal muscles and plays a crucial role in regulating protein metabolism. Although predominantly found in the nervous system, the synthesis of L-carnosine was initially observed in primary cultures of brain cells obtained from newborn mice, as well as in a glioma cell line derived from rats (Berezhnoy et al., 2019). Furthermore, it has been established that L-carnosine exhibits a neuroprotective effect in an experimental model of Parkinson's disease (Zhao et al., 2017). Moreover, L-carnosine has been found to inhibit cell death and modulate mitochondrial energy metabolism in neuron cultures subjected to oxygen-glucose deprivation (Ouyang et al., 2016). In a study conducted by Schalch Junior et al. (2022), it was reported that prenatal protein-energy supplementation throughout the entire duration of pregnancy in beef cattle leads to alterations in serum L-carnosine levels in the resulting calves. Notably, the most substantial differences in L-carnosine levels were observed in the plasma of calves subjected to protein-energy supplementation during pregnancy. Recently, Suwanvichanee et al. (2022) investigated the effects of β-alanine and L-histidine supplementation in slow-growing chickens. Based on their findings, it was demonstrated that both β-alanine and L-histidine can effectively synthesize elevated levels of carnosine without adversely affecting meat quality. Additionally, these supplements were found to influence the secondary structures of proteins and improve meat texture. Nonetheless, a deeper understanding of the molecular mechanisms underlying carnosine synthesis in chickens is warranted in order to identify and elucidate markers that can facilitate the development of more refined nutrient selection programs (Suwanvichanee et al., 2022).

The gastrointestinal system in newly hatched chickens is not fully developed, and the lack of proper digestion and absorption can lead to increased mortality and reduced performance after hatching. By injecting peptides or proteins into the eggs, the growth performance of broiler chicks can be improved by enhancing neuronal, hormonal, or intestinal absorptive capacity (Peebles, 2018). Research has shown that injecting L-carnosine into the eggs can decrease serum glucose, TG, and LDL levels, while increasing TP and HDL levels in newly hatched chickens (Ismail and Ouda, 2020). In the diet of broiler chickens, the addition of L-carnitine (100–600 mg/kg) can increase TP levels and decrease serum cholesterol, TG, LDL, and glucose levels (Ismail and Ouda, 2020). Furthermore, supplementing the diet with L-carnosine (100–400 mg/kg) can increase the antioxidant capacity of the liver, serum, and breast muscle (Cong et al., 2017). Carnosine possesses exceptional antioxidant properties, and administering it orally can reduce the production of malondialdehyde in the serum, liver, and brain of chickens (Aydın et al., 2016). A recent study by Promkhun et al. (2023), found that supplementing the diet with β-alanine and L-histidine positively correlated with various metabolomic and biochemical compounds in the jejunum of slow-growing chickens, such as amide I, amide II, creatine, tyrosine, valine, isoleucine, and aspartate.

Based on the available literature, there are no reports on the in ovo injection of L-carnosine, making it difficult to compare the results. Therefore, we examined reports on the in ovo injection of L-histidine and β-alanine. It has been reported that injecting β-alanine into the eggs can enhance hatching characteristics, carcass yield, and meat quality in broiler chickens (Atan and Kop-Bozbay, 2021). Similarly, injecting L-histidine (0.55 mg) into the eggs on d 7 of incubation has been shown to improve hatchability, intestinal development, and growth performance (Xu et al., 2019). L-carnosine has been found to protect the brain against ischemic brain injury and prevent cognitive disorders in Alzheimer's disease when supplemented with L-histidine (Solis et al., 2015). Additionally, L-carnosine supplementation (1.5 g per d) has been shown to ameliorate neurological symptoms in Parkinson's disease (Boldyrev et al., 2008). It has also been reported that L-carnosine improves mental fatigue, memory, and motor function (Baraniuk et al., 2013). These findings suggest that in ovo feeding of L-carnosine can be beneficial for neonatal broiler chickens without any adverse effects.

Houjeghani et al. (2018) reported that dietary supplementation of L-carnosine (500 mg/kg) resulted in decreased fasting glucose and HbA1c levels, as well as increased serum TG and HDL levels in patients with type 2 diabetes, without affecting IL-6 and IL-1β levels. L-carnosine, known for its fat-burning properties, can reduce fat and subcutaneous adiposity. Soliman et al. (2007) found that L-carnosine (100 and 200 mg/kg) decreased hyperglycemia in diabetic rats. Our findings are consistent with previous reports. L-carnosine can decrease sympathetic nervous system activity, increase insulin secretion, and suppress glucagon secretion (Nagai et al., 2003). Pretreatment with an H3 receptor antagonist reversed the effect of L-carnosine on blood sugar, suggesting the involvement of the histaminergic mechanism (Yamano et al., 2001). Additionally, L-carnosine has been reported to have a TG-lowering effect. Several mechanisms have been proposed to explain the beneficial effects of L-carnosine, including its ability to inhibit protein glycation and reverse glycated protein through a translocation mechanism, thereby preventing the formation of advanced glycation end-products (Houjeghani et al., 2018). Adipokines play a crucial role in the pathophysiology of metabolic syndromes, where patients exhibit elevated levels of adiponectin and leptin. Interestingly, the administration of L-carnosine did not have any impact on the levels of adiponectin and leptin in these patients. It is possible that other hormones, such as ghrelin and glucagon-like-peptide 1, are involved in the activity of L-carnosine (Al-Sawalha et al., 2019). However, due to the limitations of the current study, we were unable to determine the role of adiponectin, leptin, ghrelin, and glucagon-like-peptide 1, as well as the underlying molecular mechanisms for the observed results. Despite the differences in the metabolic regulation systems between avian and mammalian species, further research is warranted in order to elucidate the mechanisms of action behind the observed findings.

## DISCLOSURES

No potential conflict of interest was reported by the authors.
